# Phenolic Exudation Control and Indirect Somatic Embryogenesis of Garlic-Fruit Tree (*Malania oleifera* Chun & S.K. Lee)—An Endangered Woody Tree Species of Southeastern Yunnan Province, China

**DOI:** 10.3390/plants14142186

**Published:** 2025-07-15

**Authors:** Rengasamy Anbazhakan, Xin-Meng Zhu, Neng-Qi Li, Brihaspati Poudel, Jiang-Yun Gao

**Affiliations:** Institute of Biodiversity, School of Ecology and Environmental Sciences, Yunnan University, Kunming 650500, China

**Keywords:** phenolic exudation, carbon source, *Malania oleifera*, somatic embryogenesis, nervonic acid, endemic tree, reintroduction

## Abstract

*Malania oleifera* Chun & S.K. Lee, an endemic monotypic species that belongs to the family Olacaceae, is under continuous pressure of decline owing to several ecological and physiological factors. The present study aimed to establish an efficient in vitro protocol for callus-mediated indirect somatic embryogenesis in *M. oleifera* by alleviating tissue browning. Internodes and leaves obtained from seedlings were used as explants. Antioxidant pre-treatment (ascorbic acid, AA) followed by different carbon sources (sucrose, maltose, glucose, and fructose) and plant growth regulators in various concentrations and combinations were employed in Woody Plant Medium (WPM) to alleviate explant browning and induce callus formation from the explants. AA pre-treatment and subsequent culture on maltose at a concentration of 116.8 mM were optimal for controlling phenolic exudation on >90% of both explants. The highest responses of 53.77% and 57.43% for embryogenic calli were induced from internode and leaf explants, respectively. The highest responses, 85.22% and 93.80%, were observed for somatic embryos that matured into the globular, heart-shaped and torpedo stages at different percentages on NAA 2.5 mg/L in combination with BA 1.0 mg/L for both explants. The matured somatic embryos were finally germinated at a maximum concentration of GA_3_, 2.0 mg/L. All plantlets were successfully hardened and acclimatized under culture room conditions and then transferred to the greenhouse. The current study suggests an efficient protocol for indirect somatic embryogenesis by alleviating phenolic exudation from the explants of *M. oleifera*. This first successful report of in vitro culture establishment in *M. oleifera* may offer an effective alternative measure to conserve this species and provide a system for analyzing bioactive chemicals and for use in the oil industry.

## 1. Introduction

The exploration and utilization of non-timber biological resources derived from woody trees have long been the focus of forestry studies. Oilseeds originating from woody plants have high potential to meet the increasing requirement for vegetable oils for use in food or industrial applications [1]. *Malania oleifera* Chun & S.K. Lee belongs to the family Olacaceae, and it is an endemic tree species found in southeast Yunnan and west Guangxi provinces, China. Its seeds have been utilized to make edible oils and have been eaten by local people for several decades. The seeds contain >60% oil with the highest-known proportion (>55%) of nervonic acid [2,3,4]. In addition, several other constituents, such as benzaldehyde, benzyl alcohol, and benzoic acid, have also been recorded in all parts of the plant. Nervonic acid, a monounsaturated fatty acid with a very long chain, is a major component of mammalian brain nerve fibers and nerve cells. It has potential commercial value and medicinal value in healing and treating brain nerve injuries, neurological disorders and associated diseases, multiple sclerosis symptoms, Alzheimer’s disease, adrenoleukodystrophy, and Zellweger syndrome [5,6,7,8,9].

This plant is currently listed as vulnerable (VU) according to the International Union for Conservation of Nature (IUCN)’s Red List category and is also recorded in the “Red Data Book of Chinese Plants” [10,11,12]. Over-exploitation, together with a small and decreasing population and narrow distribution, renders this plant under serious threat [13,14]. A recent genomic study on the diverse populations of *M. oleifera* has predicted a serious loss of suitable habitat of up to 98.79% in the future based on ecological niche modeling [15]. They also report that the accumulation of deleterious mutations by inbreeding, anthropogenic disturbance, and increased annual temperatures have been responsible for habitat fragmentation and endangerment of the species. Hence, an urgent need for ex situ conservation of this threatened species is recommended.

Natural and conventional propagation are difficult due to various physiological factors, such as the large seed size, short life span of the seeds, challenging natural seed germination, lower rate of pollen germination, hemi-parasitic behavior of the roots, and susceptibility to root rot [16,17]. Recent reports have recorded problems with the germination of mature seeds of *M. oleifera* due to their dormant nature and the long duration, 5–7 months, required to germinate under a moist sand layer, characterized by low seedling emergence and uneven germination [18]. A frequent high rate of seedling death has also been recorded, challenging and limiting its widespread use.

In vitro plant tissue culture has been a solution to overcome various challenges in the propagation of tree species. Somatic embryogenesis is one of the techniques used in plant tissue culture, especially for woody plants, to produce a large number of elite clones and to achieve the propagation of high-value trees [19]. There is growing interest in developing somatic embryogenesis systems for medicinal and woody plants [20,21]. Several influential factors, such as explant type, culture media, plant growth regulators (PGRs), and culture conditions, affect somatic embryo induction [19]. Nevertheless, tissue browning is a major challenge in raising woody plants under in vitro conditions due to high levels of secondary metabolites, such as tannins and other phenolic compounds [22]. Moreover, several influential factors are responsible for tissue browning, both at internal and external levels. Externally, the carbon source is one of the principal components of the tissue culture medium, serving as an osmotic agent, and plays a significant role in callus induction and regeneration [23]. To date, no study has been recorded on in vitro propagation of *M. oleifera*, which may owe to tissue browning due to a large amount of secondary metabolites [4]. An optimal source of carbon and plant growth regulators (PGRs) could help to mitigate tissue browning and callus induction in *M. oleifera*. Considering the importance of the plant for conservation and its poor record of conventional propagation, the current study aimed to develop a simple and efficient protocol for controlling phenolic exudation and callus-mediated indirect somatic embryogenesis from internode and leaf explants of *M. oleifera.*

## 2. Results

Preliminary screening of internode and leaf explants cultured on WPM without AA pre-treatment and sugars showed complete (100%) necrosis in 24 h. However, 0.5% AA pre-treatment for 15 min resulted in 50–65% explants being free of phenolic exudation and browning, with nearly 50% of explants completely green on the same medium.

### 2.1. Effect of Carbon Sources in Controlling Phenolic Exudation from Explants and Callus Culture

**Figure 1:**
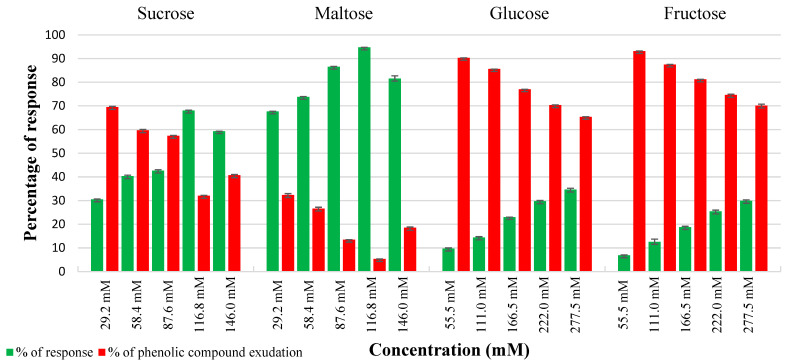
Impact of various carbon sources on phenolic exudation from internode explants on WPM supplemented with different carbon sources, after 4 weeks of culture. Values represent means ± S.E.

**Figure 2:**
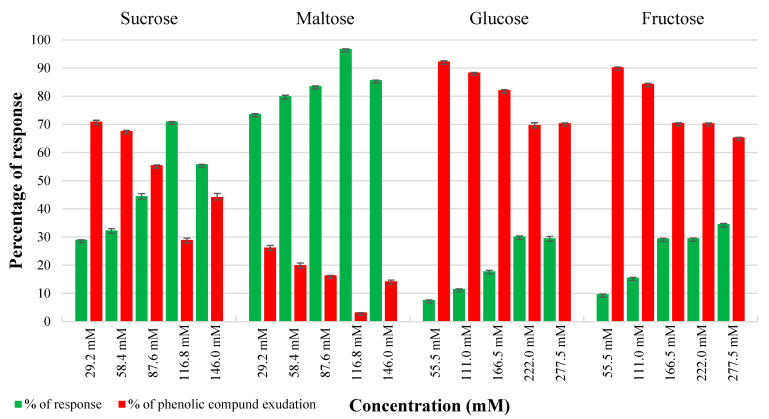
Effect of different carbon sources on phenolic exudation from leaf explants on WPM supplemented with different carbon sources, after 4 weeks of culture. Values represent means ± S.E.

In vitro cultures of leaf and internode explants on basal WPM without any carbon source, served as a control, encountered nearly 50% browning and necrosis of tissues. In contrast, both internode and leaf explants showed comparable embryogenic callus induction responses on all the carbon sources treated. The most effective carbon source among the various carbon sources examined was determined to be 116.8 mM maltose, followed by sucrose (116.8 mM) to control phenolic exudation from explants. Maltose at both lower and higher molar concentrations had a negligible effect on tissue browning compared to other carbon sources. The highest response of 94.7% and 96.77% phenolic-free green friable embryogenic callus was obtained from internode and leaf explants from maltose 116.8 mM, respectively (Figure 1 and Figure 2). Sucrose at 116.8 mM demonstrated optimal callus growth (68%) in internodes with a significant quantity of phenolic exudation (32%) (Figure 1), whereas leaf explants showed a better callus response (71%) with reduced phenolic exudation (29%) at this same optimal level. In contrast, fructose and glucose showed higher phenolic exudation in both explants. Noticeably, phenolic exudation was higher in internodes than leaf explants from both treatments. Maltose was found to be crucial in reducing phenolic exudation from the explants. However, maltose at lower and above optimal levels showed tissue browning at a negligible rate of 5–15% explants.

### 2.2. Embryogenic Callus Induction

**Figure 3:**
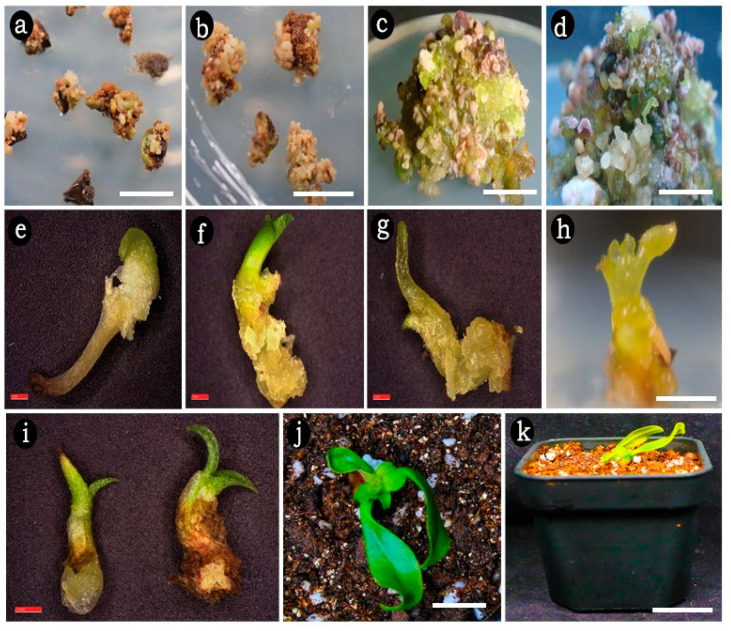
Embryogenic callus induction and somatic embryo formation in *M. oleifera*. (**a**) Induction of embryogenic callus, (**b**) proliferation of embryogenic callus, (**c**,**d**) somatic embryo induction and maturation (globular and heart-shaped embryos), (**e**) torpedo stage somatic embryo with bipolar structure, (**f**–**i**) somatic embryo germination into plantlets, (**j**,**k**) acclimatized plantlets. Scale bars: (**a**–**d**,**h**) = 5 mm, (**e**–**g**,**i**) = 1 mm, (**j**) = 1 cm, (**k**) = 2 cm.

In the current study, the rate of embryogenic callus response depended on the various concentrations and type of auxins tested. Within one week of transferring the explants to WPM supplemented with NAA or 2,4-D, the cultured leaf and internode explants began to expand, and after four weeks, embryogenic callus clumps had formed with subsequent formation of spherical embryos in both types of explants. This finding indicates that both auxins can initiate and promote the early stages of somatic embryogenesis. On WPM supplemented with NAA at and above 2.5 mg/L, over 20 to 30 globular embryos were formed after two months of culture (Table 1 and Figure 3a,b). The response rates were 49.43% and 57.43%, with an average of 21.03 and 31.88 somatic embryos per explant, respectively. In contrast, callus response and the mean number of somatic embryos were significantly lower on 2,4-D. This study demonstrated that spherical embryos were significantly higher (>20) at concentrations above 2.0 mg/L on both auxins whereas good quality green friable embryogenic callus with globular embryos was recorded only at NAA 2.5 mg/L. Hence in the subsequent experiments, WPM with NAA (2.5 mg/L) was employed with cytokinins to test their ability in the maturation of somatic embryos.

### 2.3. Somatic Embryo Maturation

**Figure 4:**
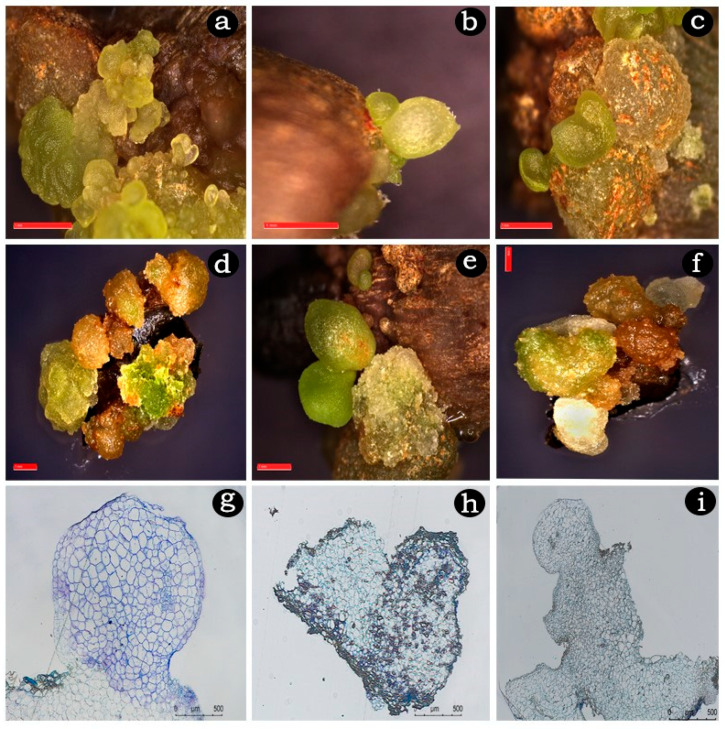
Different developmental stages of somatic embryos and histological analysis of *M. oleifera*. (**a**–**c**) Different developmental stages of somatic embryos from internode explant derived callus, (**d**–**f**) different developmental stages of somatic embryos from leaf explant derived callus, (**g**) histology of globular embryo, (**h**) histology of heart-shaped embryo, (**i**) histology of cotyledonary stage embryos showing bipolar structures derived from torpedo-shaped embryo. Scale bars: (**a**–**f**) = 1 mm, (**g**–**i**) = 500 μm.

Combinations of NAA (2.5 mg/L) with cytokinins demonstrated a significant increase in embryo formation and development of different embryogenic stages (Table 2 and Figure 3c,d). Among the cytokinin combinations, NAA with BA (1.0 mg/L) produced the highest frequencies of 85.22% and 93.80% embryo induction and the highest number of spherical and heart-shaped embryos (85.77 and 116.25, and 4.29 and 37.58) as well as torpedo-shaped embryos (1.94 and 9.55) per callus culture from both explants after 12 weeks of culture (Table 2 and Figure 4a–f). Other cytokinin combinations, NAA with TDZ or KIN resulted in a competitively lesser embryogenic response and only up to globular and heart-shaped embryos. They yielded a higher frequency of 69.55% and 76.04% and the maximum number of spherical and heart-shaped embryos of 54.41 and 82.17, and 2.51 and 9.75 for both explants. Both explants at lower cytokinin combinations failed to promote embryo maturation beyond the globular stage where torpedo-shaped embryos were completely absent.

### 2.4. Effect of GA_3_ on Germination of Somatic Embryos

Torpedo-stage embryos from all the treatments were detached from the friable embryogenic callus and inoculated onto WPM supplemented with BA (1.0 mg/L) and GA_3_ (1.0 to 3.0 mg/L) to promote embryo germination (Table 3 and Figure 3e,f). The rate of embryo germination and the recovered number of plantlets were assessed after 6 weeks of culture (Figure 3g–i). The well-germinated rooted plantlets were carefully removed, thoroughly washed with sterile water, and transferred to plastic pots (6.5 cm in diameter) filled with a mixture of red soil, sand, and coco pith at a 1:1:1 (*w*/*w*/*w*) ratio and covered with polyethylene bags (Figure 3j,k). These pots were initially maintained in the culture room for two weeks and then transferred to the green-house condition with a maximum survival rate of 50%.

### 2.5. Histological Analysis

Histological examinations of somatic embryos revealed a distinct bipolarity beginning at the globular stage (Figure 4g). Vascular differentiation became clearly visible as it progressed from the heart-shaped stage (Figure 4h). The shoot meristems were analyzed during the transition from the late torpedo stage to the cotyledonary stage (Figure 4i).

## 3. Discussion

Forest resources are under serious concern due to over-exploitation by industrialization and urbanization. The increasing demand for forest produce for the growing global population has become a serious concern, for which proper management and utilization of forest products are highly recommended [24]. Plant tissue culture has been an effective tool for the production of quality planting stocks in a limited time. However, tissue browning in vitro is a serious concern with woody plants, influenced by several internal factors such as species, genotypes, physiological status of the explants, and external factors such as, explant treatment, medium ingredients, carbon source, growth regulators and environmental conditions [22]. Of the two categories of tissue browning, non-enzymatic browning which is not associated with phenolic compounds can be alleviated by frequent subcultures, whereas enzymatic browning involves the oxidation of phenolics by the enzymes polyphenol oxidase (PPO) and peroxidase (POD) leading to the formation of brown quinones that inhibit the growth and development of cultures [25]. Any stress or damage to plant tissues, especially in tree species, during in vitro culture can disrupt the cellular phenolics to react with the oxidative enzyme, thereby producing tissue browning [26].

Antioxidants such as ascorbic acid, citric acid, glutathione, L-cysteine, silver nitrate, sodium thiosulfate, etc., play a vital role in scavenging reactive oxygen species (ROS) and protecting cells from damage. Ascorbic acid is a strong antioxidant capable of decreasing oxidative tissue browning by scavenging oxygen radicals produced during abiotic stress conditions, including explant injury, at varying levels as recorded in many plants [27,28]. Antioxidants including ascorbic acid either as media supplements or external pre-treatments have been recorded to protect the cultures or explants from oxidative stress damage and prevent tissue browning under normal culture conditions in several species such as *Musa* spp. cv. Kanthali, *Solanum surattense*, *Sapindus mukorossi*, *Glycine max*, etc. [27,29,30,31]. In our study, 0.5% ascorbic acid pre-treatment for 15 min was shown to be effective in preventing nearly 65% of explants from oxidative damage, compared to the control where complete necrosis of the explant was recorded. An earlier study in *Glycine max* may explain the current observation with the ascorbic acid treatment [30]. They have reported that, although the external supply of ascorbic acid was unstable and has a short lifetime, the uptake and transport within the roots of *Glycine max* took only a few hours under ideal conditions. Complete saturation of the cut ends and possible uptake of the ascorbic acid solution by the explants could be the reason for the positive effect in the present study.

A suitable carbon source in optimal concentration is said to be essential to regulate cell proliferation and differentiation, especially for callus culture [22]. In order to mitigate tissue browning from leaf and internode, various sugars in different concentrations were assessed by autoclaving with the WPM. Sucrose, a disaccharide, is a common source of carbon, most frequently used by autoclaving in plant tissue culture media. Earlier reports on in vitro studies have also suggested that both filter-sterilized and autoclaved disaccharide and monosaccharide sugars have shown similar results besides being sensitive to heat [32,33]. A recent work on *Hancornia speciosa* cell-suspension culture has recorded the better performance of autoclaved glucose and sucrose, by their increased rate of absorption and higher osmotic potential on the medium [34]. Of the various sugars tested, maltose showed an effective response in reducing tissue browning compared to sucrose, glucose and fructose. Maltose, a disaccharide that remained intact in the medium, was effective in inhibiting browning of plant cells [35]. It has a slower rate of extracellular hydrolysis than sucrose, leading to delayed uptake and hydrolysis. Maltose’s capacity to modulate phenolic release into the culture medium can be attributed to this function. Maltose was employed as an effective carbon source in the callus culture of the *Narasimha* cotton cultivar to limit the emission of excessive phenols [36]. Similarly in other cotton species employed maltose confirmed no tissue browning at any stage of callus culture [37,38]. Maltose has also been recorded to enhance plant regeneration compared to sucrose, even though phenolic segregation was not seen in *Urochloa brizantha* [39]. The results of the current investigation support the above statement for callus culture of *M. oleifera*, in which 116.8 mM of maltose was effectively used as the only carbon source until the development of callus to avoid phenolic exudation.

Sucrose is a commonly used sugar in different media for the growth and development of several plants. However, the response may vary depending on the concentration and the species. Several studies have shown the incidence of phenolic exudation with sucrose in a dose-dependent manner leading to necrosis in species such as *Gossypium hirsutum* [38] *Salix myrsinifolia* [40], and *Origanum vulgare* [41]. In contrast, a reverse trend of lowering explant browning was recorded from lower to higher concentrations in *M. oleifera*. Both glucose and fructose failed to inhibit tissue browning in this study. Previous records on *Taxus brevifolia* confirmed reduced callus growth with severe tissue browning possibly due to increased secondary metabolite production in cultures treated with glucose and higher amount of fructose [42,43]. The autoclaving of sugars, especially glucose and fructose, along with the media components can be degraded into toxic components. The extent of degradation to toxic levels depends on the concentration of sugars, volume of solution, pH, container, storage conditions, temperature, and duration of autoclaving [44,45]. Both glucose and fructose have been recorded to produce acidic toxic compounds, glucose degradation products (GDPs) and 5-(hydroxymethyl)-2-furaldehyde (HMF) mainly at high temperatures and extensive autoclaving periods of over 20 min [45,46,47]. Based on the findings of [46], a considerable number of GDPs and HMF formed during the current autoclaving conditions (121 °C for 15 min) and further storage at 25 °C may be responsible for the diminishing effect of glucose and fructose in callus induction.

Embryonic development in angiosperms can be divided into three phases: cell proliferation, morphogenesis and maturation [48]. The types of auxins or cytokinins or their combinations and appropriate concentrations vary from species to species and must be carefully elaborated to obtain the best medium for the induction of somatic embryogenesis [49]. Unlike cytokinins, exogenous auxins can delay polyphenol biosynthesis and reduce tissue browning [50]. The present study in *M. oleifera* is in concordance with the above statement that tissue browning was comparatively low in all the auxins and their cytokinin combinations (Table 1 and Table 2). In this study, we report a successful protocol for the in vitro propagation of *M. oleifera* through indirect somatic embryogenesis for the first time by alleviating tissue browning. This promising technique offers an efficient option for the large-scale production of regenerated plantlets, which has the potential to enhance conservation efforts for valuable and endangered species. Given that *M. oleifera* is classified as an endangered and endemic species, there is an urgent need to develop a long-term conservation strategy for it.

In this study, leaf explants outperformed internode explants in terms of generating somatic embryogenesis. The best results were obtained with NAA compared to 2,4-D, which was used for both explants. In studies on other woody plants, the frequency of somatic embryogenesis ranged from 4.9% to 63% [1,51,52], and the frequency of SE in *M. oleifera* was high in the studies. Similarly, NAA has been reported to affect somatic embryonic development in *Ranunculus sceleratus* [53]. The study on *Elaeis guineensis* shows that NAA has limited efficacy in promoting somatic embryo development compared to other plant growth regulators [54]. In addition, the plant growth regulator picloram has also been used to enhance somatic embryogenesis in different plants compared to NAA in *Eucalyptus* species [55]. In this study, we discovered that the combination of NAA and BA produced synergistic effects, leading to the most favorable results for somatic embryogenesis in *M. oleifera* when both the leaf and the internode were used as explants. Similarly, the combination of NAA and BA has been reported in other woody plants, such as *Punica granatum* and *Paeonia ostii* [56,57]. The type of explant, its age, genotype, nutritional status and interactions between endogenous phytohormones and external plant growth regulators can strongly influence somatic embryogenesis [58,59]. Therefore, the selection of PGR types and concentrations is an important prerequisite for the development of somatic embryos in woody plants. We are pleased to announce that we have successfully generated plantlets from mature somatic embryos (in the globular, heart, and torpedo stages) sourced from leaf and internode explants of *M. oleifera*. After being transferred to the germination medium, cotyledonary embryos exhibited successful plantlet conversion. The rate of somatic embryo conversion into plantlets was significantly influenced by the addition of gibberellic acid (GA_3_) to the germination medium. Consequently, within two months of culture, the embryos established complete plantlets and developed both shoot and root axes. The crucial stimulatory effect of GA_3_ on somatic embryo germination was confirmed in *Plectranthus bourneae* [60]. The germination of somatic embryos was significantly enhanced by the addition of BA (0.5 mg/L) at a concentration of 0.5 mg/L, in conjunction with varying concentrations of GA_3_. Similarly, *Euonymus alatus* was found to accumulate BA alongside GA_3_, resulting in higher rates of somatic embryo germination in woody plants [61]. The combined effects of BA and GA_3_, along with other plant growth regulators, have also been reported to enhance somatic embryo development and plantlet conversion in *Plectranthus bourneae* [60] and *okra* [62].

## 4. Materials and Methods

### 4.1. Explant Source

*Malania oleifera* seedlings maintained in the greenhouse of Yunnan University were used to collect the explants. The seedlings were originally raised from seeds collected from wild trees around Tianfang Village, Shuguang Town, Guangnan County, Wenshan Zhuang, and Miao Autonomous Prefecture, Yunnan Province, China. Twenty numbers of one-year old seedlings of *M. oleifera* were maintained under controlled conditions of 27 ± 3 °C and a relative humidity (RH) of 70%.

### 4.2. Explant Surface Sterilization and Preparation

Freshly collected internode (IN) and leaf (L) explants were kept in a conical flask containing sterile water and a few drops of Tween 20, and vortexed in an orbital shaker at 60 rpm for 20 min. Then, the explants were washed under running tap water for 30 min and surface sterilized with 70% ethanol for 30 s, followed by 0.1% HgCl_2_ for 3–5 min, and finally washed with sterile distilled water three times. The surface-sterilized explants were treated with 0.5% (*w*/*v*) ascorbic acid (AA) for 15 min. The AA solution was prepared by dissolving 2.5 g of ascorbic acid in sterile distilled water following filter sterilization in a 0.22 μm filter unit and made into a final volume of 500 mL using sterile distilled water. Then, the pre-treated explants were cut into pieces of about 1 cm (internode) and 0.5 cm^2^ (leaf) and inoculated on the Woody Plant Medium (WPM). The whole process was carried out under the laminar air-flow chamber in aseptic conditions.

### 4.3. Basal Media and Culture Conditions

WPM was used as the basal nutrient medium for all the experiments. WPM was supplemented with 100 mg/L meso-inositol and 0.8% agar. The pH of the medium was adjusted to 5.7 ± 0.2 prior to autoclaving at 121 °C for 15 min. All the cultures were maintained in the culture room at 25 ± 2 °C under a 16 h photoperiod unless otherwise mentioned, provided with cool white fluorescent lamps at 35 μE m^−2^ s^−1^, and >85% relative humidity. All the chemicals used in the study were purchased from Solarbio Life Sciences, Beijing, China.

### 4.4. Influence of Carbon Source in Controlling Phenolic Exudation from Explants

Different carbon sources such as sucrose and maltose were tested at concentrations ranging from 55.5 mM to 277.5 mM, and glucose and fructose were tested at concentrations ranging from 29.2 mM to 146 mM (all equivalent to 10–50 g/L) to evaluate their effect on controlling phenolic exudation from the explants. All media with varying sugar concentrations were adjusted to a pH of 5.7 ± 0.02 and autoclaved at 121 °C and 1.05 kg cm^−2^ pressure for 15 min. The autoclaved media were left in the culture room conditions overnight and then inoculated immediately. The carbon source that resulted in the lowest phenolic exudation and maintained good explant health was selected for further callus induction treatments.

### 4.5. Induction of Embryogenic Callus

The surface-sterilized internode and leaf explants were inoculated onto WPM containing 116.8 mM maltose augmented with auxins, 1-naphthaleneacetic acid (NAA) and 2,4-Dichlorophenoxyacetic acid (2,4-D) separately each at concentrations of 1.0, 1.5, 2.0, 2.5, and 3.0 mg/L for embryogenic callus induction. The cultures were initially incubated in the dark at 23 ± 2 °C for 2 weeks and then shifted to culture room condition. The rates of embryogenic callus (EC) and somatic embryos (SEs) were calculated as the percentage of explants producing at least one globular somatic embryo out of the total number of explants inoculated.

### 4.6. Effect of PGRs on Somatic Embryo Induction and Maturation

The EC masses were split into pieces of about 0.5 g fresh weight and transferred into WPM augmented with NAA (2.5 mg/L) in combination with cytokinins such as thidiazuron (TDZ), 6-Benzylaminopurine (BA), and kinetin (KIN) at 0.5, 1.0, 1.5, and 2.0 mg/L for somatic embryo induction and maturation. Each treatment consisted of five replicates, each containing about 30 ECs. The cultures were maintained in 16/8 h (light/dark) photoperiod at 23 ± 2 °C. All cultures were subcultured every 4 weeks. After 12 weeks of culture, the rates of somatic embryo proliferation, stages and number of SEs were recorded.

### 4.7. Somatic Embryo Germination

The mature somatic embryos at the torpedo stage were transferred to WPM augmented with 1.0 mg/L BA in combination with varying concentrations of GA_3_ (1.0 to 3.0 mg/L) to assess embryo germination. The frequency of somatic embryo development and the number of plantlets were recorded after 6 weeks of culture.

### 4.8. Resin Sections and Histological Observation for Light Microscopy

Embryogenic callus and SEs were collected at various stages for histological analysis. Samples were fixed overnight in a formalin-acetic acid-alcohol fixative. Following dehydration in an ethanol series (50%, 75%, 85%, 95%, and 100% *v*/*v*; 2 h at each concentration) at room temperature, the samples were infiltrated with a 2:1 (*v*/*v*) mixture of ethanol and LR White acrylic resin (L9774; Sigma-Aldrich, St. Louis, MO, USA) for 1 h, followed by a 1:2 (*v*/*v*) mixture for 2 h, and then 100% LR White acrylic resin overnight at 4 °C. The samples were subsequently embedded in LR White acrylic resin. Semi-thin sections (3 μm) were stained with 1% (*w*/*v*) toluidine blue O (TBO) and examined under a light microscope (Leica Microsystems, Wetzlar, Germany) [63].

### 4.9. Experimental Design and Statistical Analysis

Both explants were cultured on 15 × 90 mm Petri plates containing approximately 30 mL of culture medium. All the experiments were carried out in a completely randomized design. Each treatment had a minimum of ten replicates, and each experiment was repeated thrice, except for somatic embryo induction and maturation. All the cultures were subcultured every 3 weeks on the same medium. The results of carbon source treatment were recorded after 2 weeks, and callus treatments were recorded after 30 days of culture. All the data were subjected to analysis of variance (ANOVA), and significant differences between the treatments were analyzed using Duncan’s multiple range test (DMRT) at *p* < 0.05 using statistical software IBM SPSS Statistics, version 25.0 (Armonk, NY, USA).

## 5. Conclusions

This study provides the first experimental evidence of controlling phenolic exudation in *M. oleifera*. Furthermore, the *M. oleifera* cultivar commonly cultivated in the western Guangxi Province and south east Yunnan Province of China is recalcitrant to in vitro treatment, making it necessary to develop an appropriate in vitro methodology to achieve high frequency regeneration. To date, there are no reports on the effect of carbon sources on the control of phenolic exudation, callus induction and somatic embryogenesis in *M. oleifera.* The present investigation offers a practical solution to the tricky problem of phenolic exudation in callus. Ascorbic acid pre-treated explants cultured on WPM supplemented with maltose 116.8 mM and exogenous PGRs such as NAA 2.5 mg/L, BA 1.0 mg/L and GA_3_ 2.0 mg/L, either alone or in combinations were found to mitigate explant browning and callus-mediated somatic embryogenesis. The combination of PGRs significantly enhanced the potential for plant regeneration from somatic embryos via indirect somatic embryogenesis. The economic and medicinal significance of *M. oleifera* will be considered in future initiatives aimed at tissue culture techniques for conservation, mass clonal propagation, production of bioactive compounds, and genetic transformation purposes.

## Figures and Tables

**Table 1:** Embryogenic callus induction from internode and leaf explants of *M. oleifera*, on WPM supplemented with auxins, after 8 weeks of culture.

Auxins	Percentage of Embryogenic Callus (%)		Number of Somatic Embryos	
	Internode	Leaf	Internode	Leaf
NAA				
1.0	26.54 ± 0.12 ^i^	33.04 ± 0.38 ^i^	4.61 ± 0.17 ^h^	8.47 ± 0.06 ^i^
1.5	33.83 ± 0.24 ^g^	37.66 ± 0.16 ^g^	9.06 ± 0.36 ^f^	14.84 ± 0.30 ^g^
2.0	53.77 ± 0.19 ^a^	45.66 ± 0.10 ^f^	14.06 ± 0.40 ^e^	19.59 ± 0.90 ^e^
2.5	49.43 ± 0.10 ^b^	57.43 ± 0.6 ^a^	21.03 ± 0.30 ^c^	31.88 ± 0.72 ^b^
3.0	45.69 ± 0.06 ^d^	51.07 ± 0.39 ^c^	27.65 ± 0.11 ^a^	36.66 ± 0.12 ^a^
2,4-D				
1.0	23.65 ± 0.34 ^j^	30.69 ± 0.41 ^j^	6.65 ± 0.06 ^g^	10.44 ± 0.04 ^g^
1.5	27.62 ± 0.13 ^h^	34.66 ± 0.24 ^h^	9.54 ± 0.07 ^f^	15.63 ± 0.12 ^f^
2.0	39.16 ± 0.31 ^f^	54.61 ± 0.05 ^b^	15.65 ± 0.22 ^d^	20.58 ± 0.13 ^d^
2.5	46.69 ± 0.09 ^c^	48.61 ± 0.07 ^d^	21.65 ± 0.06 ^c^	27.79 ± 0.13 ^c^
3.0	43.29 ± 0.09 ^e^	46.76 ± 0.11 ^e^	26.76 ± 0.11 ^b^	32.51 ± 0.13 ^b^

Values represent means ± S.E. Values followed by the same letter are not significantly different at *p* ≤ 0.05 according to DMRT.

**Table 2:** Effect of NAA (2.5 mg/L) in combinations with different cytokinins on somatic embryo maturation from internode and leaf derived embryogenic callus, after 12 weeks of culture.

Cytokinins	Percentage of Response (SE)		Average Number of Somatic Embryos					
	Internode	Leaf	Internode			Leaf		
			Globular	Heart	Torpedo	Globular	Heart	Torpedo
TDZ								
0.5	50.73 ± 0.20 ^j^	58.21 ± 0.56 ^i^	44.51 ± 0.33 ^k^	0.00 ± 0.00 ^g^	0.00 ± 0.00 ^e^	56.22 ± 0.33 ^j^	0.00 ± 0.00 ^j^	0.00 ± 0.00 ^e^
1.0	59.57 ± 0.30 ^g^	67.65 ± 0.16 ^g^	47.62 ± 0.08 ^j^	0.00 ± 0.00 ^g^	0.00 ± 0.00 ^e^	67.36 ± 0.30 ^i^	7.44 ± 0.19 ^g^	0.00 ± 0.00 ^e^
1.5	69.55 ± 0.16 ^d^	76.04 ± 0.19 ^d^	54.41 ± 0.09 ^i^	2.51 ± 0.26 ^c^	0.00 ± 0.00 ^e^	82.17 ± 0.81 ^g^	9.75 ± 0.18 ^f^	0.00 ± 0.00 ^e^
2.0	63.48 ± 0.22 ^e^	74.69 ± 0.07 ^e^	60.62 ± 0.70 ^g^	0.00 ± 0.00 ^g^	0.00 ± 0.00 ^e^	86.33 ± 0.44 ^f^	11.48 ± 0.09 ^e^	0.00 ± 0.00 ^e^
BA								
0.5	61.70 ± 0.25 ^f^	73.04 ± 0.39 ^f^	78.69 ± 0.10 ^c^	2.33 ± 0.01 ^d^	1.25 ± 0.31 ^d^	98.56 ± 0.12 ^d^	25.66 ± 0.05 ^d^	4.29 ± 0.03 ^d^
1.0	85.22 ± 0.36 ^a^	93.80 ± 0.31 ^a^	85.77 ± 0.16 ^b^	4.29 ± 0.05 ^a^	1.94 ± 0.02 ^c^	116.25 ± 3.64 ^a^	37.58 ± 0.04 ^a^	9.55 ± 0.57 ^a^
1.5	79.66 ± 0.11 ^b^	86.78 ± 0.06 ^b^	95.66 ± 0.57 ^a^	3.44 ± 0.21 ^b^	2.33 ± 0.06 ^b^	104.85 ± 0.70 ^c^	31.22 ± 0.38 ^c^	7.71 ± 0.08 ^b^
2.0	72.71 ± 0.22 ^c^	80.55 ± 0.77 ^c^	79.35 ± 0.35 ^c^	2.57 ± 0.06 ^c^	2.88 ± 0.12 ^a^	109.18 ± 0.36 ^b^	34.83 ± 0.06 ^b^	6.55 ± 0.06 ^c^
Kin								
0.5	49.59 ± 0.04 ^k^	57.47 ± 0.12 ^i^	58.62 ± 0.03 ^h^	0.00 ± 0.00 ^g^	0.00 ± 0.00 ^e^	78.65 ± 0.10 ^h^	00 ± 0.00 ^j^	00 ± 0.00 ^e^
1.0	55.85 ± 0.16 ^i^	61.74 ± 0.26 ^h^	67.72 ± 0.07 ^e^	1.28 ± 0.04 ^f^	0.00 ± 0.00 ^e^	86.51 ± 0.60 ^f^	00 ± 0.00 ^j^	00 ± 0.00 ^e^
1.5	63.52 ± 0.22 ^e^	67.77 ± 0.11 ^g^	77.77 ± 0.06 ^d^	1.95 ± 0.06 ^e^	0.00 ± 0.00 ^e^	94.33 ± 0.48 ^e^	2.51 ± 0.03 ^i^	00 ± 0.00 ^e^
2.0	58.62 ± 0.21 ^h^	61.11 ± 0.11 ^h^	66.84 ± 0.03 ^f^	2.28 ± 0.9 ^d^	0.00 ± 0.00 ^e^	87.84 ± 0.34 ^f^	5.65 ± 0.06 ^h^	00 ± 0.00 ^e^

Values represent means ± S.E. Values followed by the same letter are not significantly different at *p* ≤ 0.05 according to DMRT.

**Table 3:** Effects of WPM containing BA (1.0 mg/L) with different concentrations of GA_3_ on torpedo somatic embryos germination, after 6 weeks of culture.

GA_3_ (mg/L)	SEs Germination (%)		Mean Number of Somatic Plantlets Recovered	
	Internode	Leaf	Internode	Leaf
1.0	9.43 ± 0.05 ^e^	11.51 ± 0.12 ^d^	2.65 ± 0.10 ^e^	3.47 ± 0.13 ^e^
1.5	11.50 ± 0.10 ^d^	13.54 ± 0.12 ^c^	3.65 ± 0.11 ^d^	4.72 ± 0.10 ^d^
2.0	16.54 ± 0.06 ^a^	17.47 ± 0.03 ^a^	5.87 ± 0.02 ^a^	7.62 ± 0.13 ^a^
2.5	14.40 ± 0.15 ^b^	15.54 ± 0.06 ^b^	5.44 ± 0.23 ^b^	7.09 ± 0.01 ^b^
3.0	12.51 ± 0.07 ^c^	13.54 ± 0.12 ^c^	4.88 ± 0.05 ^c^	5.69 ± 0.13 ^c^

Values represent means ± S.E. Values followed by the same letter are not significantly different at *p* ≤ 0.05 according to DMRT.

## Data Availability

The datasets supporting the conclusions are not included in the article.
